# Supplementary material for: Ligand distances as key predictors of pathogenicity and function in NMDA receptors
Source: Hum Mol Genet. 2024 Nov 13;34(2):128–39. doi: 10.1093/hmg/ddae156 (PMC11780861; doi:10.1093/hmg/ddae156)
Supplement: Supplemental_Notes_FigS1_TabS1_TabS2_ddae156 [file supplemental_notes_figs1_tabs1_tabs2_ddae156.pdf]

Ligand Distances as Key Predictors of Pathogenicity and Function in NMDA Receptors.

SUPPLEMENTARY MATERIAL

Supplementary Notes

ML computation and cross-validation

The binary classifiers of this study were trained with random forests and implemented through the RandomForestClassifier function of the scikit-learn python package. A combination of 16 estimators, ranging from 10 and 500, and eight different maximum depth parameters, ranging from 2 to 9, were explored. A 5-fold cross-validation was adopted for both the pathogenicity predictor and the Increase/Decrease predictor. Each cross-validation comprises a training, a validation and a test set. The best parameters were chosen as the ones which maximize the Matthews correlation coefficient (MCC) on the validation set. The performances reported in the results section are computed on the test set. Given that distance features are only based on the 3D position of the variant (independently of the substituted residue), two different variants in the same position will have the same distance features. To avoid the risk of overfitting, we pooled variants at the same position (therefore with the same distance features) in the same cross-validation set. For the predictors only based on distance features, only one variant has been retained for each residue position.

Performance measures for the binary classifiers

The classifiers were evaluated using the following indexes. For each prediction, the classification for each binary classifier (Pathogenic/Benign and Increase/Decrease) is made at the threshold 0.5. In all

the performance measures, TP (true positives) are correctly predicted variants of the first class (Pathogenic or Increase), TN (true negatives) are correctly predicted variants of the second class (Benign or Decrease), FP (false positives) are variants of the second class (Benign or Decrease) that are predicted to belong to the first class (Pathogenic or Increase), and FN (false negatives) are variants of the first class (Pathogenic or Increase) that are predicted to belong to the second class (Benign or Decrease). Predictor performance was evaluated using the following metrics: true positive and negative rates (*TPR, TNR*), positive and negative predicted values (*PPV, NPV*), and overall accuracy (Q_2)

$$PPV = \frac{TP}{TP + FP} \quad TPR = \frac{TP}{TP + FN}$$

$$NPV = \frac{TN}{TN + FN} \quad TNR = \frac{TN}{TN + FP}$$

[Eq. 1]

$$Q_2 = \frac{TP + TN}{TP + FP + TN + FN}$$

We computed the Matthew's correlation coefficient MCC (Eq. 2) as:

$$MCC = \frac{TP \times TN - FP \times FN}{\sqrt{(TP + FP)\ (TP + FN)\ (TN + FP)\ (TN + FN)}}$$

[Eq. 2]

We also calculated the area under the receiver operating characteristic (ROC) curve (AUC), by plotting the True Positive Rate as a function of the False Positive Rate and the Area and the Precision Recall Curve (AUP) at different probability thresholds.

Supplementary Figures

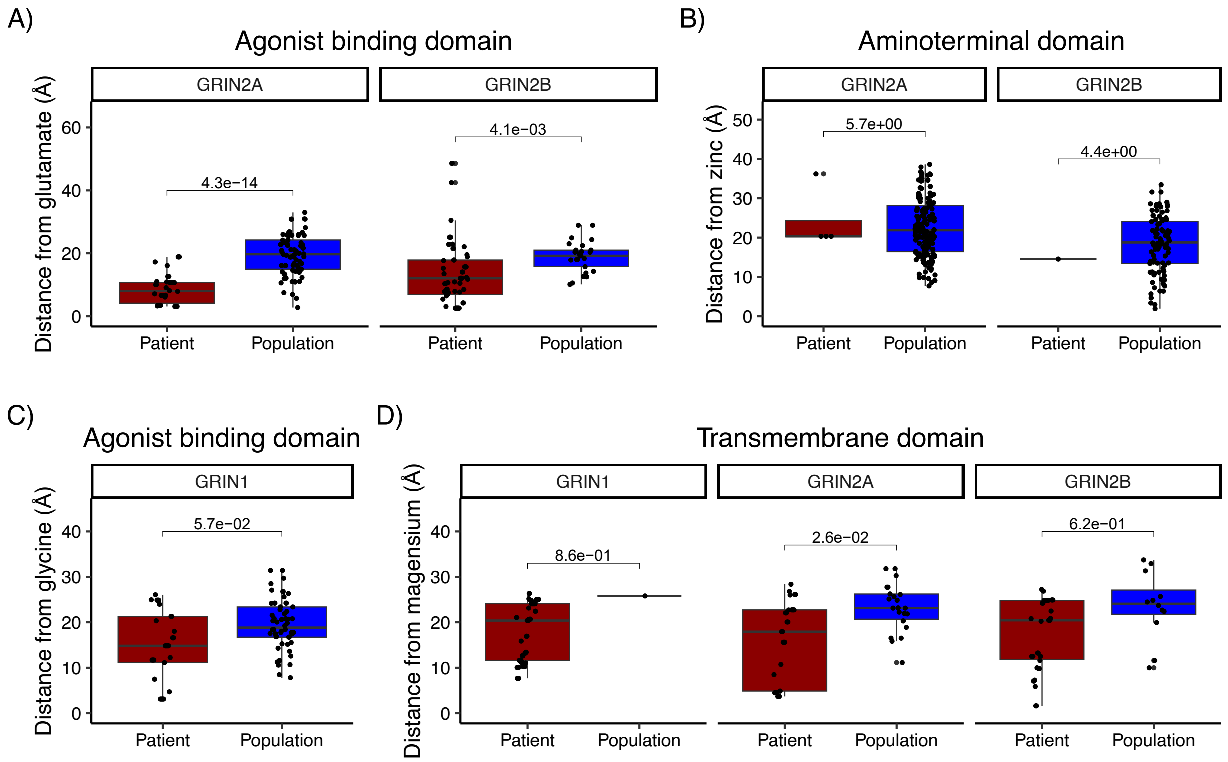

Supplementary Figure S1 Variant distance from NMDAR ligands significantly differs between patient and population variants in the GRIN-genes. Boxplot of the distances of patient and population variants from four different NMDAR ligands: **A)** glutamate, **B)** Zn^{2+}, **C)** glycine, and **D)** Mg^{2+}. Only variants that are located in the same domain where the ligand is bound are shown and the distance is only computed for the protein subunits that are involved into the ligand binding. To quantify the differences in the distances to the ligands, we performed the Wilcoxon-rank sum test and corrected for eight test using Bonferroni correction.

Supplementary Tables

Table S1 List of the considered distance, biophysical, and evolutionary features for missense variants

Feature	Short name	Description
		**Distance features**
Distance from glutamate	dist_glu	Distance (Å) of the wild-type residue from glutamate ligand using all-atoms.
Distance from glycine	dist_gly	Distance (Å) of the wild-type residue from glycine ligand using all-atoms.
Distance from Mg^{2+}	dist_mg	Distance (Å) of the wild-type residue from Mg^{2+} ion using all-atoms.
Distance from Zn^{2+}	dist_zn	Distance (Å) of the wild-type residue from Zn^{2+} ion using all-atoms.
Distance from prem axis	dist_pore	Distance (Å) of the wild-type residue from the pore axis using all-atoms.
Distance from membrane	dist_mem	Distance (Å) of the wild-type residue from membrane using all-atoms.
		**Biophysical features**
Relative solvent accessibility	RSA	Relative solvent accessibility of the wilt-type residue computed through the DSSP program (Kabsch and Sander 1983).
Hydrophobicity difference	KD	Hydrophobicity score accounting for the difference in hydrophobicity between wildtype and mutate residue as in Montanucci *et al.*, 2019.
Energy difference	E3D	Difference in the interaction energy between the wild-type and mutate residue with their structural environment (5 Å sphere) as in Montanucci *et al.*, 2019.
		**Evolutionary features**
Conservation difference	S_BLOSUM62	Difference between the wild-type and variant residue in the BLOSUM62 substitution matrix as in Montanucci *et al.*, 2019.
EVE evolutionary model	EVE	Score from the deep learning based and unsupervised EVE model

Table S2 Description of the input features for each pathogenicity and functional effect predictor.

Predictor	Input features*
	Pathogenicity Predictor (PP)
PP-dist	dist_glu, dist_gly, dist_mg, dist_zn, dist_pore
PP-evo	S_BLOSUM62, EVE
PP-biophys	RSA, KD, E3D
PP-dist&evo	dist_glu, dist_gly, dist_mg, dist_zn, dist_pore, S_BLOSUM62
	Functional Predictor (FP)
FP-dist	dist_glu, dist_gly, dist_mg, dist_zn
FP-evo	S_BLOSUM62, EVE
FP-biophys	RSA, KD, E3D
FP-dist&evo	dist_glu, dist_gly, dist_mg, dist_zn, S_BLOSUM62, EVE

* Short names of the features are derived from Table 1.
